# Vitamin D Inhibits Myogenic Cell Fusion and Expression of Fusogenic Genes

**DOI:** 10.3390/nu12082192

**Published:** 2020-07-23

**Authors:** Tohru Hosoyama, Hiroki Iida, Minako Kawai-Takaishi, Ken Watanabe

**Affiliations:** 1 Department of Regenerative Medicine, National Center for Geriatrics and Gerontology, Obu, Aichi 474-8511, Japan; hirokida@ncgg.go.jp (H.I.); mkawai@ncgg.go.jp (M.K.-T.); 2 Department of Orthopaedic Surgery, Nagoya University Graduate School of Medicine, Nagoya, Aichi 466-8560, Japan; 3 Department of Bone and Joint Disease, National Center for Geriatrics and Gerontology, Obu, Aichi 474-8511, Japan; kwatanab@ncgg.go.jp

**Keywords:** vitamin D, cell fusion, fusogenic gene, hypertrophy, sarcopenia

## Abstract

Vitamin D, a fat-soluble vitamin, is an important nutrient for tissue homeostasis and is recently gaining attention for its role in sarcopenia. Although several studies have focused on the role of vitamin D in muscle homeostasis, the molecular mechanism underlying its action on skeletal muscle remains unclear. This study investigated the role of vitamin D in myogenesis and muscle fiber maintenance in an immortalized mouse myogenic cell line. A high concentration of active vitamin D, 1α,25(OH)_2_D_3_, decreased the expression of myogenic regulatory factors (MRFs), *myf5* and *myogenin* in proliferating myoblasts. In addition, high concentration of vitamin D reduced myoblast-to-myoblast and myoblast-to-myotube fusion through the inhibition of *Tmem8c* (myomaker) and *Gm7325* (myomerger), which encode muscle-specific fusion-related micropeptides. A similar inhibitory effect of vitamin D was also observed in immortalized human myogenic cells. A high concentration of vitamin D also induced hypertrophy of multinucleated myotubes by stimulating protein anabolism. The results from this study indicated that vitamin D had both positive and negative effects on muscle homeostasis, such as in muscle regeneration and myofiber maintenance. Elderly individuals face a higher risk of falling and suffering fractures; hence, administration of vitamin D for treating fractures in the elderly could actually promote fusion impairment and, consequently, severe defects in muscle regeneration. Therefore, our results suggest that vitamin D replacement therapy should be used for prevention of age-related muscle loss, rather than for treatment of sarcopenia.

## 1. Introduction

Skeletal muscle is one of largest tissues in the body, and its volume and function are well maintained during life until middle age. However, from middle age, the maintenance system for muscle homeostasis gradually declines, resulting in sarcopenia, an age-related muscular phenotype with evident loss of muscle mass and strength [1]. Sarcopenia is currently recognized as an independent age-related muscle disorder and is diagnosed in about 5–10% of individuals over 65 years of age [2,3]. Although the mechanisms behind the onset and progress of sarcopenia have not been fully understood, it is believed to be a result of several factors such as diseases, decreased caloric intake, mitochondrial dysfunction, and increase in oxidative stress. Furthermore, humoral changes resulting from decreased circulation of some vitamins, cytokines, and growth factors are also known to be contributing factors in the occurrence of sarcopenia in elderlies [4]. Among humoral factors, the level of vitamin D in circulation is considered to be a sarcopenia-related factor; this is because there is some evidence suggesting that a decrease or deficiency in vitamin D in circulation in the blood of most elderlies results in muscle loss and decline of muscle strength [5]. However, the relationship between vitamin D and sarcopenia is yet to be proven scientifically, and further scientific research is needed for clarification.

Vitamin D, a fat-soluble vitamin, is endogenously produced in the skin on exposure to sunlight and can also be obtained from foods and dietary supplements. Firstly, vitamin D is metabolized to 25-hydroxyvitamin D (25OHD) in liver, and then 25OHD is further hydroxylated in the kidneys to the active form 1,25-dihydroxyvitamin D (1,25(OH)_2_D). In liver, vitamin D is metabolized to 25OHD by cytochrome P450 oxidases, CYP2R1 and CYP27B1, and 25OHD is metabolized to 1,25(OH)_2_D by CYP27B1 in the kidneys, and 25OHD and 1,25(OH)_2_D are catabolized by CYP24A1. Although 25OHD and 1,25(OH)_2_D circulate with vitamin D binding protein (DBP), the blood level of 25OHD is higher than the active form vitamin D (1,25(OH)_2_D) [6,7]. An active form of vitamin D binds to vitamin D receptor (VDR), which is a nuclear receptor and heterodimerize with retinoid X receptor (RXR) following binding to active vitamin D, and then the VDR-RXR heterodimer binds to vitamin D response element (VDRE) in the promoter region of target genes [8]. Although one of the most known effects of vitamin D is on the bone and in mineral homeostasis, it is thought that vitamin D also plays a role in myogenesis, including in the proliferation and differentiation of myoblasts [9,10,11]. For instance, proliferation of chick myoblasts is inhibited by treatment with vitamin D, and vitamin D downregulates the expression of myogenin, which is a necessary factor in the terminal differentiation of murine myoblast [9,10,12,13]. Additionally, vitamin D treatment stimulates the Akt-mTOR-mediated pathway and varies the diameter of murine myotubes through the regulation of an anabolic and a catabolic pathway [14]. Taken together, it is expected that vitamin D signaling has certain effects on myogenesis and muscle fiber maintenance through multiple pathways. However, the effects of vitamin D in skeletal muscle are still controversial because both positive and negative results of its effects have been reported [15]. For example, high-dose supplementation of vitamin D causes inadequate differentiation of myogenic cells in regenerating muscle [16] and increases the risk of falls [17,18].

In this study, we examined the effect of vitamin D on myogenesis and muscle fiber maintenance of immortalized mouse myogenic cells. Consistent with previous studies, the expression of muscle-related factors (MRFs) and terminal differentiation were inhibited in proliferating mouse myoblasts by a high concentration of vitamin D. Intriguingly, a high concentration of vitamin D inhibited both myoblast-to-myoblast and myoblast-to-myotube fusions, which are necessary for the formation of multinucleated mature myotubes. In this study, on the one hand, a decrease in the expression of fusogenic genes, *Tmem8c* (myomaker) and *Gm7325* (myomerger, also known as myomixer or minion), was also observed in vitamin D-treated myoblasts, suggesting that vitamin D inhibited the differentiation of myogenic cells through the regulation of muscle-specific fusogenic micropeptides. On the other hand, vitamin D stimulated an anabolic pathway in multinucleated myotubes resulting in hypertrophy. Taken together, these results suggest that vitamin D possesses dual roles in myogenesis and in muscle fiber homeostasis.

## 2. Materials and Methods

### 2.1. Cell Culture

The immortalized mouse myogenic cell clone Ric10 and human myogenic cell clone Hu_5_KD_3_ were kindly gifted from Dr. Naohiro Hashimoto [19,20]. The Ric10 and Hu_5_KD_3_ cells were plated on a type I collagen-coated cell culture dish and cultivated in 20% FBS/DMEM supplemented with 2% Ultroser G serum substitute (Pall Corp, Port Washington, NY, USA). For proliferation assay, 3 × 10^4^ cells were seeded onto the cell culture dish (24-well cell culture dish, 1.8 cm^2^ culture area) and cultivated with 1α,25(OH)_2_D_3_ (1 μM; Cayman Chemical, Ann Arbor, MI, USA) for 24 h. At 18 h cultivation, 5-ethynyl-2′-deoxyuridine (EdU; Thermo Fisher Scientific, Waltham, MA, USA) was added into the medium for pulse chase of myoblast proliferation, and cells were further incubated for 6 h. For differentiation, the medium was changed to 2% HS/DMEM when the Ric10 myoblasts reached confluence. At 0 and 24 h of differentiation condition, vitamin D (1 μM) was added into the medium to study its effect on myotube formation, and cells were collected after 48 h of vitamin D treatment for gene or protein expression analysis. In addition, vitamin D was added into the medium at 48 h of differentiation and cultivated for another 48 h to study its effect on anabolic/catabolic pathways. PA452 (2.5 μM; Tocris Bioscience, Bristol, UK), which is an RXR antagonist, was co-administered with vitamin D in the fusion experiments.

### 2.2. Quantitative RT-PCR

Total RNA was extracted from Ric10 myoblasts or myotubes at appropriate timing, and cDNA was synthesized using a SuperPrep II Cell Lysis Kit (TOYOBO, Osaka, Japan). Polymerase chain reaction was performed using a CFX96 real-time PCR detection system (Bio-Rad, Santa Rosa, CA, USA) and PowerUp SYBR Green Master Mix (Thermo Fisher Scientific). Primer sequences are listed in Table S1.

### 2.3. Western Blotting

Protein was extracted from Ric10 myoblasts or myotubes with SDS-HBS (1% SDS/150 mM NaCl/10 mM HEPES, pH 7.4). After heat denaturing and sonication, the protein extract was mixed with the Laemmli sample buffer and boiled at 95 °C, for 5 min. Twenty micrograms of each sample were used for polyacrylamide gel electrophoresis, and then electroblotted using PVDF membrane. Following blocking with 5% skim milk/PBS-Tween20 (PBST), for 1 h at room temperature, the membrane was incubated with the primary antibody overnight at 4 °C. The following primary antibodies were used in this study: phosphorylated Smad2/3, Smad2/3, phosphorylated Foxo1a, phosphorylated Foxo3a, phosphorylated p70S6K, p70S6K, phosphorylated Akt, Akt, cleaved caspase-3, β-actin (1:1000, Cell Signaling Technologies, Danvers, MA, USA), Bax (1:500, Merck Millipore, Bedford, MA, USA), Myf5, and myogenin (1:200, Santa Cruz, CA, USA). The secondary antibodies used in this study were HRP-conjugated anti-rabbit IgG and anti-mouse IgG (1:4000, Cell Signaling Technologies).

### 2.4. Immunocytochemistry

For immunocytochemistry, cells were crosslinked with 4% paraformaldehyde (PFA), for 15 min at room temperature. After washing with phosphate-buffered saline (PBS), cells were blocked with 4% normal goat serum/0.1% Tween 20/PBS, for 1 h at room temperature, and then incubated overnight with anti-myosin heavy chain antibody (MF20, 1:100; eBioscience, San Diego, CA, USA) at 4 °C. After washing with PBS, cells were further reacted with goat anti-mouse IgG AlexaFluor 488 or 594 (1:400, Abcam, Cambridge, UK), for 1 h at room temperature. DAPI in anti-fading reagent was used for nuclear staining. The myotube diameter was measured in 449 and 537 MHC^+^ myotubes for control and vitamin D-treated groups, respectively. In this experiment, multinucleated myotubes were defined as ≥3 nuclei of MHC^+^ cells.

### 2.5. Lipid and Content Mixing Assay

To analyze the effect of vitamin D on cell fusion, a fusion synchronization approach, previously described [21,22,23], was utilized. Briefly, Ric10 myoblasts were labeled with fluorescent lipid Dil-Red (lipid probe) or membrane-permeant Green CMFDA cell tracker (content probe), respectively, and Ric10 myotubes were labeled with Green CMFDA cell tracker. To assay myoblast-to-myoblast fusion, equal numbers of labeled Ric10 myoblasts (2 × 10^4^ cells) were co-cultured under the differentiation condition with or without vitamin D for 48 h, and then cells were observed under a fluorescence microscope. The number of labeled myotubes was counted, and the ratio of dual-labeled myotubes (red^+^/green^+^) was calculated from the total myotubes including single (red^+^ or green^+^) and dual-labeled myotubes. To assay myoblast-to-myotube fusion, red labeled-Ric10 myoblasts (2 × 10^4^ cells) were added into the culture including green-labeled myotubes, which were cultivated under the differentiation condition, for 48 h. After 48 h with or without vitamin D, cells were observed under a fluorescence microscope, and the ratio of green-labeled myotubes with lipid probe was calculated from total green-labeled myotubes.

### 2.6. Statistical Analysis

To quantify each experiment, at least 3 independent experiments were performed and 5–7 randomly chosen fields were imaged and cells counted for each experimental group. To determine statistical significance, a nonparametric two-tailed t-test was used for two-group comparison and one-way ANOVA for three-group comparison. Statistical analyses were performed using the GraphPad Prism 7 software. The criterion for statistical significance was *p* < 0.05.

## 3. Results

### 3.1. Vitamin D Does Not Influence the Proliferation of Mouse Immortalized Myoblasts, Whereas Some Myogenic Genes Are Inhibited

**Figure 1:**
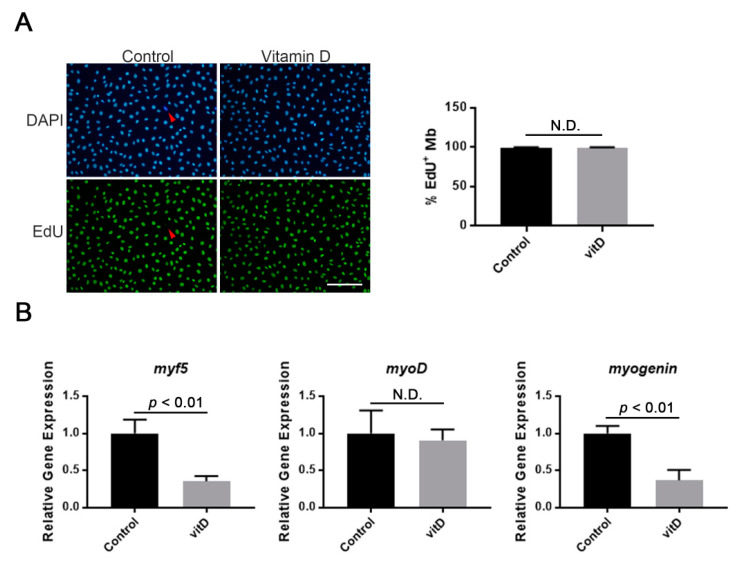
Effect of vitamin D on the proliferation and myogenic regulatory factor (MRF) expression of mouse immortalized myoblasts. (**A**) High concentration of vitamin D (vitD) did not affect myoblast proliferation. EdU was added to the growth medium 6 h before fixation in order to visualize proliferating myoblasts. Red arrowhead indicates EdU-negative myoblasts; (**B**) Expression of both *myf5* and *myogenin* was decreased in vitamin D-treated proliferating myoblasts. Scale bar = 100 μm. Mb, myoblast and N.D., no statistical significance. *p* < 0.01 is statistically significant.

The immortalized mouse myoblast cell line Ric10 was chosen to study the effect of vitamin D on proliferation and differentiation of myoblast during myogenesis because of its stability in vitro [19]. To investigate vitamin D’s action on proliferating myoblasts, a relatively high concentration of vitamin D (1 μM: 1α,25(OH)_2_D_3_) was added into the culture of proliferating Ric10 myoblasts. Following cultivation with EdU thymidine analog for 24 h, the number of EdU^+^ myoblasts was compared between vitamin D-treated and untreated groups. The results showed that there was no significant difference in EdU-positive cells, suggesting that vitamin D did not influence myoblast proliferation (Figure 1A). The effect of a high concentration of vitamin D on the expression of MRFs in proliferating myoblasts was also investigated. Consistent with previous results performed with a moderate concentration of vitamin D [10], a high concentration of vitamin D decreased the expression of muscle-specific bHLH factors, *myf5* and *myogenin*, except for *myoD* in Ric10 myoblasts (Figure 1B).

### 3.2. Vitamin D Inhibits Fusogenic Gene Expression and Myotube Formation

**Figure 2:**
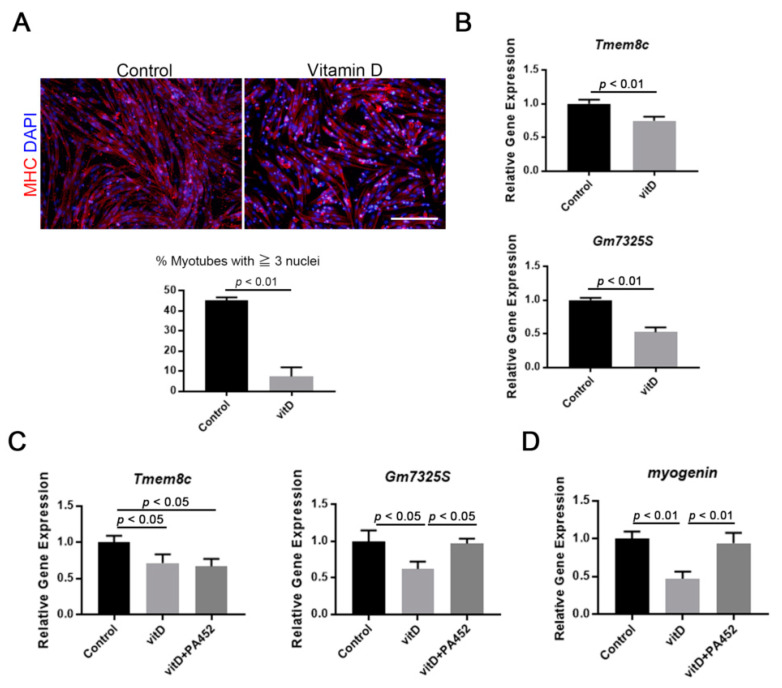
High concentration of vitamin D inhibits terminal differentiation and expression of fusogenic genes. (**A**) Formation of multinucleated myotubes (% myotubes with ≥3 nuclei in MHC^+^ cells) was suppressed by a high concentration of vitamin D; (**B**) High concentration of vitamin D decreased the expression of two fusogenic genes, *Tmem8c* (myomaker) and *Gm7325S* (myomerger-S, a short form of myomerger); (**C**) Decreased expression of fusogenic genes in vitamin D-treated myogenic cells was partially canceled by co-administration of RXR antagonist (PA452); (**D**) Vitamin D treatment caused downregulation of myogenin expression in differentiating myoblasts, and this inhibitory effect was canceled by PA452 administration. Scale bar = 100 μm. *p* < 0.01 and *p* < 0.05 are statistically significant.

The effect of vitamin D on myotube formation and fusogenic gene expression was investigated because *myogenin* expression was inhibited in proliferating myoblasts (as shown in Figure 1B). When Ric10 myoblasts reached confluence, the medium was changed from a proliferating medium to a differentiation medium, and then Ric10 myoblasts were cultivated under conditions of high vitamin D concentration, for 48 h. The results indicated that myotube formation was significantly inhibited by vitamin D treatment (Figure 2A). Two fusogenic genes, *Tmem8c* (myomaker) and *Gm7325* (myomerger), were also downregulated in vitamin D-treated Ric10 myotubes (Figure 2B and Supplemental Figure S1), and *Gm7325* and also *myogenin* expression were recovered by the administration of RXR antagonist, PA452 (Figure 2C,D). Interestingly, downregulation of fusogenic genes by vitamin D treatment was also induced in proliferating myoblasts (Supplemental Figure S1C,D). Taken together with these results, the incomplete formation of myotubes in vitamin D-treated myogenic cells could be caused by the inhibition of fusogenic gene expression, although further detailed investigations are needed.

### 3.3. Vitamin D Inhibits both Myoblast-to-Myoblast and Myoblast-to-Myotube Fusion

**Figure 3:**
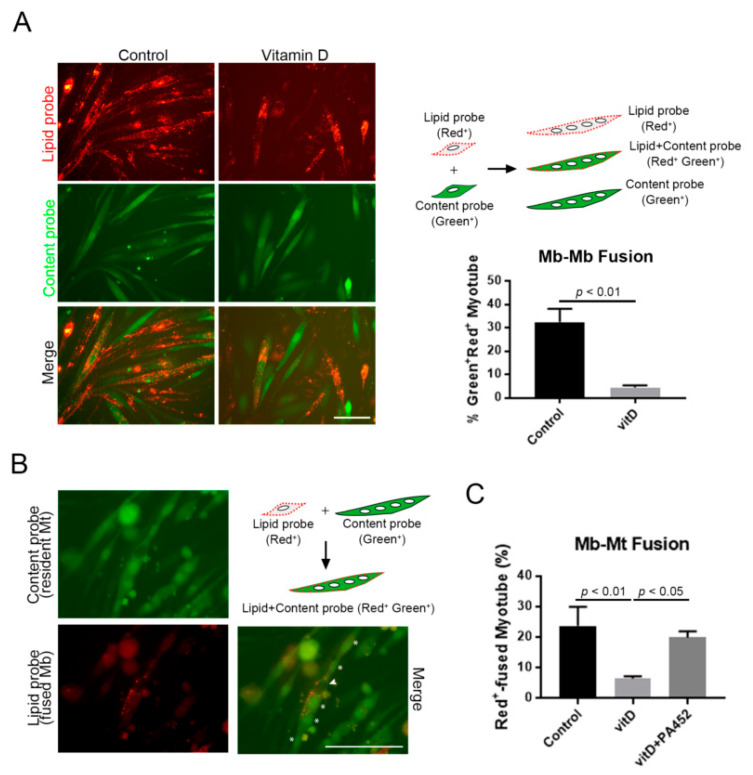
High concentration of vitamin D suppresses myogenic cell fusion. (**A**) High concentration of vitamin D inhibited myoblast-to-myoblast fusion. Two fluorescently labeled myoblasts were co-cultured for 48 h with or without high concentration of vitamin D; (**B**,**C**) High concentration of vitamin D suppressed secondary myotube formation. Mononuclear myoblasts labeled with a lipid probe (red fluorescence) were added into the culture of multinucleated myotubes labeled with a content probe (green fluorescence); then, cells were co-cultured with or without vitamin D, for 48 h, in differentiation conditions. Suppression of myoblast-to-myotube fusion was canceled by co-administration of RXR antagonist. White arrowhead and asterisks indicate expected nuclei from cells with red and green fluorescence, respectively. Mt, myotube. Scale bar = 100 μm. *p* < 0.01 and *p* < 0.05 are statistically significant.

Myotube formation is roughly divided into two steps as follows: One is myoblast-to-myoblast fusion for nascent myotubes, and the other is myoblast-to-myotube fusion to form secondary myotubes [24]; both steps are under the control of myomaker and myomerger [25,26]. This study examined whether a high concentration of vitamin D inhibited either or both steps. To check the effect on myoblast-to-myoblast fusion, Ric10 myoblasts were prelabeled with lipid or content probes, and then equal numbers of cells labeled with each color were mixed and cultured with or without vitamin D under differentiation conditions. Consequently, the ratio of double-colored myotubes calculated from all-labeled myotubes significantly decreased in the vitamin D-treated group, indicating that vitamin D inhibited fusion between myoblasts (Figure 3A). A similar result was observed also in human myogenic cells (Hu_5_KD_3_) [20] with decreased Myogenin expression (Supplemental Figure S2). Furthermore, this study investigated if a high concentration of vitamin D inhibited myoblast-to-myotube fusion. Lipid-probed myoblasts were added into the culture of content-probed myotubes, followed by further culture with or without vitamin D. Similar to the result obtained for myoblast-to-myoblast fusion, vitamin D treatment significantly decreased the number of double-colored myotubes, and this inhibition was canceled by the presence of RXR antagonist (Figure 3B,C). Thus, the incomplete formation of myotubes in vitamin D-treated myoblasts resulted from the inhibition of cell fusion caused by the suppression of fusogenic micropeptides.

### 3.4. Vitamin D Induces Myotube Hypertrophy through Stimulation of an Anabolic Pathway

**Figure 4:**
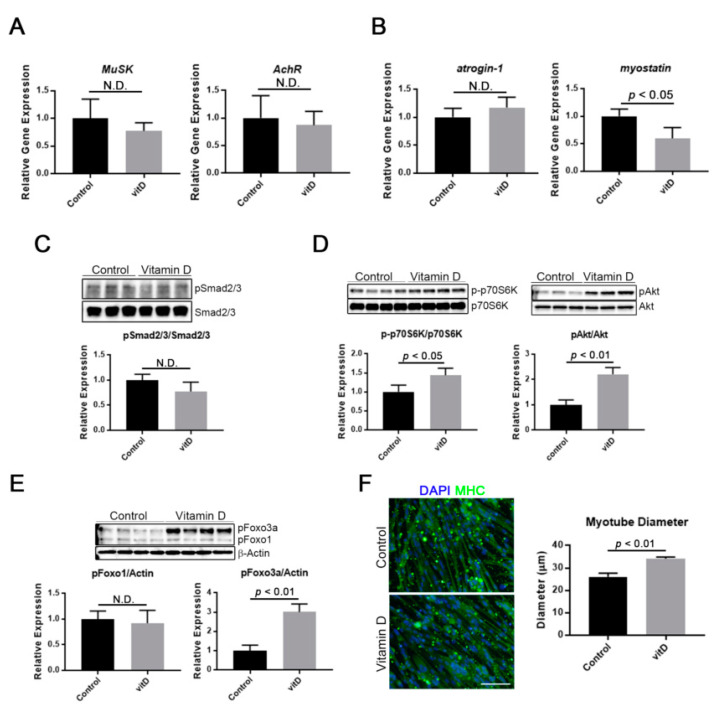
High concentration of vitamin D induces hypertrophy of myotubes via stimulation of an anabolic pathway. A high concentration of vitamin D was added to the culture of multinucleated myotubes for 48 h. (**A**) Vitamin D treatment caused no change in expression of neuromuscular junction-related genes, *MuSK* and *AChR*, in multinucleated myotubes; (**B**) A high concentration of vitamin D reduced the expression of *myostatin* but not *atrogin-1*; (**C**) Myostatin signaling was not affected by vitamin D treatment despite decreased *myostatin* expression; (**D**,**E**) High concentration of vitamin D increased the phosphorylation of Akt, p70S6K, and Foxo3a in multinucleated myotubes, indicating stimulation of the Akt-dependent anabolic pathway; (**F**) High concentration of vitamin D induced hypertrophy of myotubes (MHC^+^). Scale bar = 100 μm. N.D., no statistical significance. *p* < 0.01 and *p* < 0.05 are statistically significant.

Some reports have suggested that vitamin D was directly or indirectly involved in the maintenance of myofibers. In the present study, the effect of a high concentration of vitamin D on gene expression associated with neuromuscular junction, a catabolic, and an anabolic pathway was evaluated using Ric10 myotubes. Consequently, the results showed that vitamin D treatment did not influence the expression of neuromuscular junction-related genes (*MuSK* and *AChR*), suggesting that vitamin D was not directly involved in their expression (Figure 4A). On the other hand, vitamin D treatment had some impact on protein turnover-related pathways. In a catabolic pathway, a high concentration of vitamin D reduced the expression of *myostatin*, a negative regulator for muscle growth [27], while *atrogin-1* expression was not influenced (Figure 4B). However, vitamin D treatment did not activate the Smad pathway, which is known as the downstream of myostatin signaling [28], in Ric10 myotubes (Figure 4C). In an anabolic pathway, a high concentration of vitamin D accelerated both the Akt-p70S6K axis and the Akt-Foxo3 axis, which are protein synthesis and protein degradation inhibitory pathways [14], in myotubes (Figure 4D,E). These results indicated that vitamin D had a positive effect on protein anabolism in myotubes or myofibers. The diameters of myotubes were significantly increased by a high concentration of vitamin D (Figure 4F). We also checked cytotoxicity of vitamin D, because it was indicated that high-dose vitamin D treatment caused toxic effects [29]. As a consequence, expression of typical apoptotic-related factors, Bax and cleaved caspase 3, were not influenced in vitamin D-treated Ric10 myotubes (Supplemental Figure S3).

## 4. Discussion

In this study, we demonstrated that a high concentration of active vitamin D, 1α,25(OH)_2_D_3_, affected terminal differentiation of myoblasts. Consistent with previous studies, it was confirmed that vitamin D decreased the expression of MRFs such as Myf5 and myogenin. Previous studies have reported that this inhibitory effect on MRF expression resulted in the inhibition of terminal differentiation, because myogenin is necessary for myotube formation [30]. In this study, vitamin D inhibited myoblast-to-myoblast fusion, confirming the assertion that vitamin D inhibited terminal differentiation. It is important to note that vitamin D also inhibited the fusion of myoblasts to myotubes, indicating that vitamin D affected nascent and also mature myotube formation. Furthermore, this inhibitory effect on myogenic cell fusion was reduced by RXR antagonist, indicating the specificity of the VDR signaling. Although there are no previous studies which have explained the detailed molecular mechanisms underlying the effects of vitamin D on terminal differentiation, results from this study indicated that vitamin D inhibits the expression of two fusogenic genes, *Tmem8c* (myomaker) and *Gm7325* (myomerger), in addition to *myogenin*. These muscle specific micropeptides are necessary for myogenic cell fusion [25,26]; hence, the negative effect of vitamin D on the expression of these fusogenic genes is believed to inhibit the terminal differentiation of myogenic cells. Recently, it has been reported that myogenin directory regulates the expression of these fusogenic genes, on the one hand, by binding to E-box on their promotor regions [31], indicating that vitamin D could inhibit myogenic cell fusion through downregulation of myogenin and, consequently, fusogenic micropeptides. On the other hand, it was assumed that vitamin D inhibited the expression of myomaker and myomerger via different pathways, because, although the administration of RXR antagonist stopped the inhibitory effect of vitamin D on the expression of *Gm7325* (myomerger), it did not stop the inhibitory effect of vitamin D on the expression of *Tmem8c* (myomaker). Although the role of vitamin D in the fusion of satellite cells to multinucleated myogenic cells (myotubes and myofibers) [32] was a concern, it was not investigated in the present study. Satellite cell fusion to myofiber is important because it induces muscle hypertrophy resulting from overloaded exercise [33]; however, further investigations are needed to clarify these uncertain underlying mechanisms of vitamin D and myogenic cell fusion.

In the present study, vitamin D did not negatively affect the proliferation of myoblasts. Similar results were observed in C_2_C_12_ myoblasts [34], suggesting that vitamin D did not influence the mobility of mononuclear myoblasts. However, another study demonstrated that the proliferation and viability of C_2_C_12_ cells were influenced by vitamin D treatment [35]. Although there is no clear explanation for this discrepancy in results, it is possible that the effect of vitamin D on the proliferation of myoblasts is dose dependent and cell type dependent such as species and cell mortality. For this point, additional detailed studies using different cell types, vitamin D dosages, and time courses are needed to lead the conclusion.

Postnatal skeletal muscle is appropriately maintained in size and functions. This maintenance system is based on innervation in addition to the balance between anabolism and catabolism of muscle proteins. It has been reported that elderlies with vitamin D deficiency experienced decreased neuromuscular function [36], suggesting the importance of vitamin D in neuromuscular junction (NMJ) maintenance. A recent publication reported that vitamin D in combination with agrin administration increased acetylcholine receptor clustering and rapsyn expression in C_2_C_12_ myotubes [37]. This indicated that vitamin D and VDR signaling contributed to NMJ formation. In the present study, there was no alteration in the expression of NMJ-related genes in Ric10 myotubes subjected to a high concentration of vitamin D; thus, it was assumed that vitamin D was not directly involved in the expression of NMJ-related genes but acted synergistically with an additional component such as extracellular matrix on NMJ functions. Nonetheless, because the present study only focusing on gene expression, further investigations in protein level are necessary to clarify the importance of vitamin D and the VDR signaling in neuromuscular function and maintenance.

The balance in protein turnover is a critical factor in maintaining myofiber size, and the ubiquitin-proteasome system (UPS) is one of the major regulatory systems for protein catabolism [38]. In this study, on the one hand, expression of UPS-related gene, *atrogin-1*, was not altered by vitamin D treatment, indicating that an excess of vitamin D did not affect catabolic activities in myotubes. On the other hand, a high concentration of vitamin D accelerated the phosphorylation of Akt, p70S6K, and Foxo3a, indicating that vitamin D influenced anabolic activities in myotubes, because these molecules were involved in protein synthesis [39]. Akt phosphorylates p70S6K under the regulation of PI3K, accelerating muscle protein synthesis combined with mTOR. In addition, Akt can inactivate UPS by the induction of Foxo3a phosphorylation, inhibiting protein degradation in myogenic cells [40]. Therefore, it was assumed that a high concentration of vitamin D activated the Akt-dependent anabolic pathway, and consequently induced hypertrophy of myotubes in the present study. However, the expression *atrogin-1*, which is a downstream target of Akt-Foxo3a axis, was unchanged in response to a high concentration of vitamin D. Another pathway such as the Foxo3a-associated lysosomal pathway can be present in vitamin D-induced muscle hypertrophy [41]. Both catabolic and anabolic pathways are complicated, and further investigations are necessary to clarify the mechanism of the vitamin D signaling in muscle protein turnover.

The myostatin signaling is another muscle atrophy inducing system [28,42]. Although the relationship between vitamin D and the myostatin signaling has not been well investigated, a negative effect of vitamin D on myostatin expression has been reported in a previous study using C_2_C_12_ cells [11]. In this study, we found that *myostatin* was suppressed in vitamin D-treated Ric10 myotubes, indicating the consistency of this work with previous studies. These results suggest that vitamin D-induced *myostatin* suppression induces hypertrophy of myotubes or myofibers. However, phosphorylation of Smad2/3, which is an essential event in myostatin signaling [28], was unchanged by vitamin D treatment, indicating that in spite of decreased gene expression, the myostatin signaling pathway was not affected in myotubes treated with a high concentration of vitamin D. Considering this, the data from this study suggest that a high concentration of vitamin D predominantly accelerates the Akt-dependent pathway to induce muscle hypertrophy, but does not utilize inhibition of the myostatin-related atrophic pathway.

In this study, it was demonstrated that a high concentration of vitamin D positively affected multinucleated myotubes, suggesting that vitamin D replacement therapy is a promising approach to cure age-related muscle decline, sarcopenia. However, vitamin D treatment also affected terminal differentiation negatively. In elderlies, the risk of falling is increased because of declined-motor activity and muscle strength, causing muscle injury and, consequently, morbidity and disability. Because an event of myogenic cell fusion in postnatal muscle typically occurs in a situation of exercise-induced hypertrophy or recovery from injury, a high concentration of vitamin D could cause prohibition of myofiber reconstruction particularly during the cell fusion step. A clinical trial revealed that an annual high-dose vitamin D supplementation increased the risk of falling and fractures in elderlies [17], indicating the possibility that a high concentration of vitamin D promotes incomplete muscle regeneration after injury, and then causes decline of muscle performance in elderlies. Putting these in perspective, vitamin D has both positive and negative effects on myogenic cells; hence, vitamin D treatment should probably be used for the prevention of age-related muscle loss rather than for sarcopenia treatment. However, a number of clinical studies demonstrated an improvement of muscle strength in vitamin D-treated elderlies, suggesting that vitamin D supplementation is an effective therapeutic approach for sarcopenia [43]. In this study, we did not perform the functional study to clarify the vitamin D’s action on muscle strength. Therefore, the effect of vitamin D on muscle function needs to be clarified in further investigations.

## Supplementary Materials

The following are available online at https://www.mdpi.com/2072-6643/12/8/2192/s1, Figure S1: High concentration of vitamin D reduces expression of Myomerger isoforms, Figure S2: High concentration of vitamin D inhibits fusion of human myogenic cells, Figure S3: High concentration of vitamin D did not influence on expression of pro-apoptotic factors in multinucleated myotubes, Table S1: qPCR primer sequence.
