# Supplementary material for: Vitamin D Inhibits Myogenic Cell Fusion and Expression of Fusogenic Genes
Source: Nutrients. 2020 Jul 23;12(8):2192. doi: 10.3390/nu12082192 (PMC7469064; doi:10.3390/nu12082192)
Supplement: Supplementary file 1 [file nutrients-12-02192-s001.pdf]

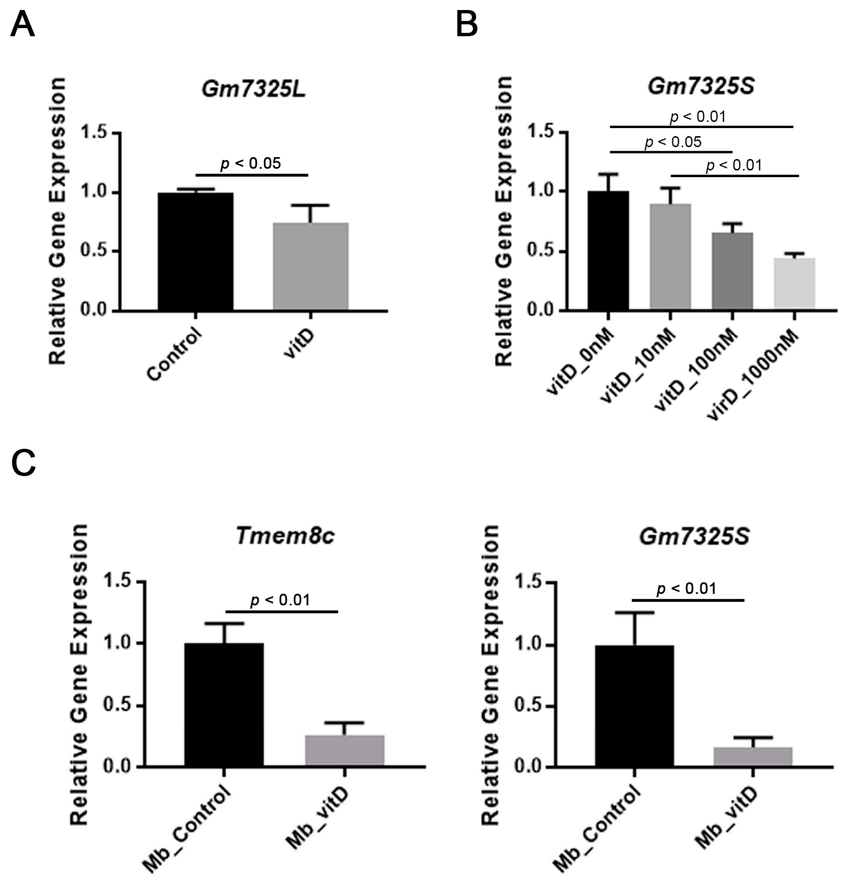

Supplemental Figure 1. High concentration of vitamin D reduces expression of Myomerger isoforms.

(**A**) High concentration of vitamin D inhibited expression of *Gm7325L* (MyomergerL, a long form of Myomerger, in myogenic cells. (**B**) Vitamin D dose-dependently decreased the expression of *Gm7325S* (MyomergerS, a short form of Myomerger). (**C, D**) High concentration of vitamin D inhibited expression of both *Tmem8c* and *Gm7352S* in proliferating myoblasts.

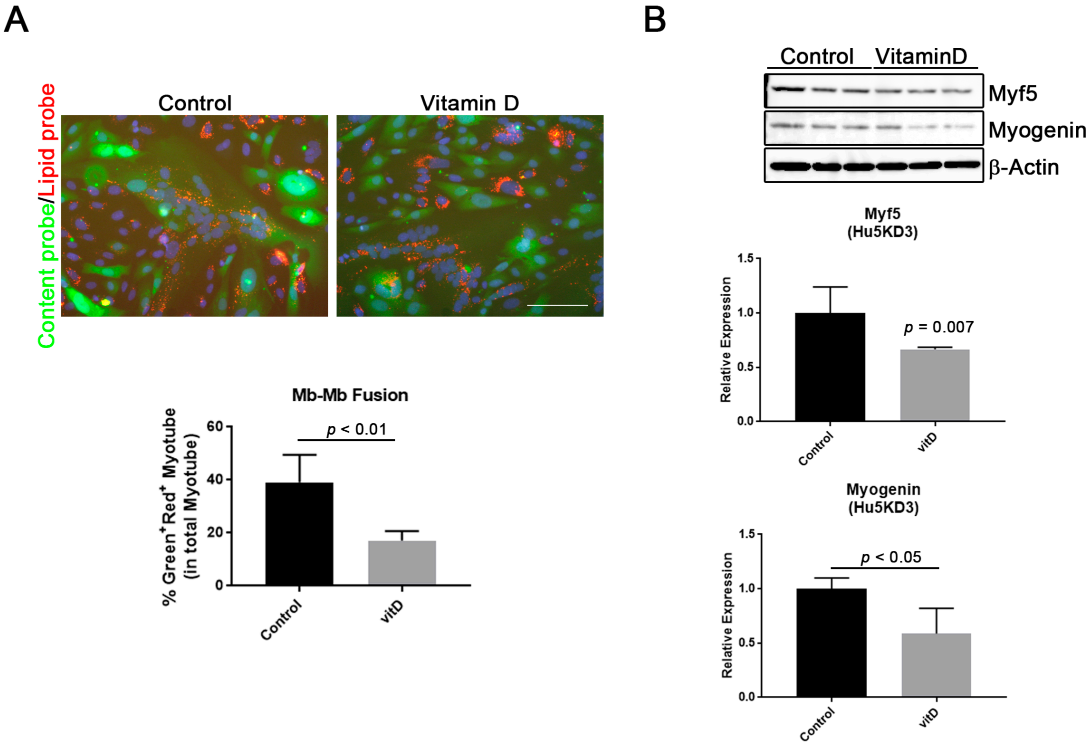

Supplemental Figure 2. High concentration of vitamin D inhibits fusion of human myogenic cells.

(**A**) Immortalized human myogenic cell line, Hu5KD3, was labeled with lipid (red) or content (green) probes and then co-cultured for 72 hours with or without vitamin D in differentiating conditions. The number of green/red double-positive multinucleated myotubes was counted to calculate the percentage of myoblast fusion. Scale bar=100 m. Mb: myoblast. (**B**) Expression of Myogenin protein was significantly decreased in differentiating human myogenic cells, while Myf5 was not altered.

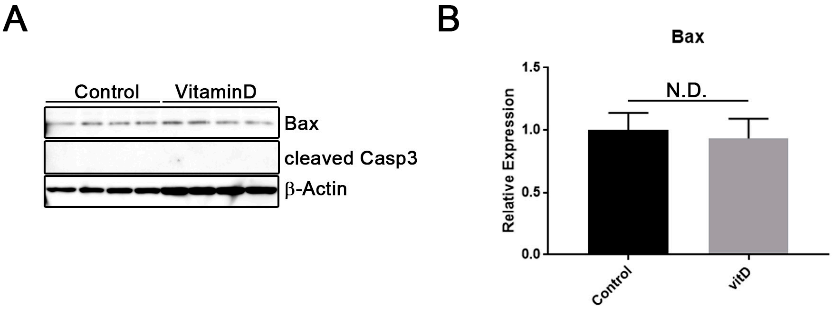

Supplemental Figure 3. High concentration of vitamin D did not influence on expression of pro-apoptotic factors in multinucleated myotubes.

(**A, B**) High concentration of vitamin D did not altered expression of Bax and cleaved caspase 3 (undetectable) proteins.

Supplementary Table S1.

pPCR Primer Sequence.

Gene	Forward (*5′-3′*)	Reverse (*5′-3′*)
myf5	*TGAGGGAACAGGTGGAGAAC*	*AGCTGGACACGGAGCTTTTA*
myoD	*AGCACTACAGTGGCGACTCA*	*GCTCCACTATGCTGGACAGG*
myogenin	*CTACAGGCCTTGCTCAGCTC*	*AGATTGTGGGCGTCTGTAGG*
atrogin-1	TGTGGGTGTATCGGATGGAG	GGCAGAGTCTTCCACAGT
myostatin	AGTGGATCTAAATGAGGGCAGT	GTTTCCAGGCGCAGCTTAC
MuSK	TGAGAACTGCCCCTTGGAACT	GGGTCTATCAGCAGGCAGCTT
AchR	CATCGAGGGCGTGAAGTACA	ATTCCTCAGCGGCGTTATTG
Tmem8c	ATCGCTACCAAGAGGCGTT	CACAGCACAGACAAACCAGG
Gm7325S	CAGGAGGGCAAGAAGTTCAG	ATGTCTTGGGAGCTCAGTCG
Gm7325L	ACCAGCTTTCATGCCAGAAG	ATGTCTTGGGAGCTCAGTCG
GAPDH	*GTGAAGGTCGGTGTGAACG*	*ATTTGATGTTAGTGGGGTCTCG*
